# Saving patients by pulling their teeth out ‐ but killing them softly afterwards with dental implants?

**DOI:** 10.1002/cre2.142

**Published:** 2018-10-26

**Authors:** Asbjorn Jokstad

As a trained and licensed doctor, society expects you to consistently provide the best care based on sound judgment of comprehensive patient histories and thorough clinical examinations, while respecting patient autonomy by encouraging shared treatment decision making. A prerequisite for the latter is that the patient receives sufficient unbiased information regarding alternative interventions, including no therapy, and their likely prognoses.

**Figure 1:**
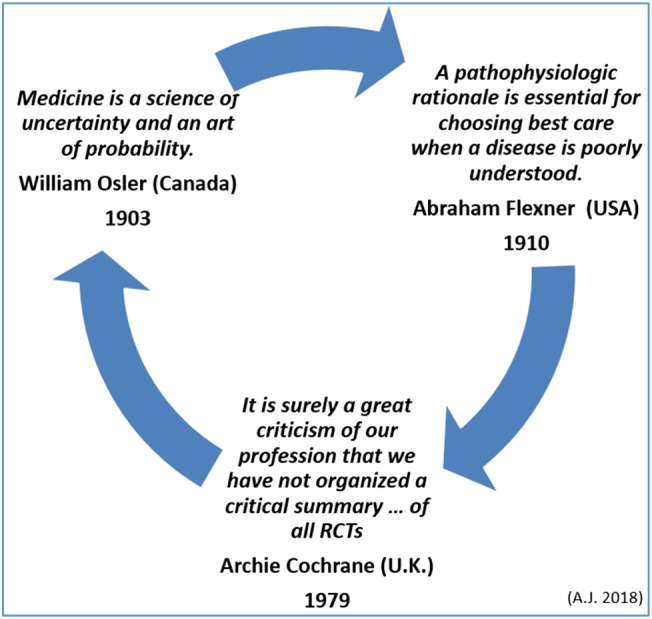
When information is unsatisfactory as a basis for providing care, healthcare providers may choose different strategies on how to advise the patient or the public

However, many times in medicine, the pertinent information is convoluted or nonexistent. In these circumstances, healthcare providers choose different strategies on how to advise the patient or the public. If any RCTs are deemed methodologically weak or fail to focus on a weighty outcome, should a particular intervention be postponed or denied or should the logic proposed by William Osler (1903), or by Abraham Flexner (1910) be followed? (Figure 1).

Infective endocarditis (I.E.) is a rare and complex disease associated with high morbidity and mortality and characterized by a multifactorial etiopathogenesis. Causative microorganisms are identified by blood culture or by polymerase chain reaction (PCR) and are in decreasing order, staphylococci, streptococci, enterococci, and HACEK organisms. Streptococci used to be the most common microorganism until about a decade ago, which is a reflection of changes of underlying causal factors for contracting I.E. (Duval, Delahaye, Alla, Tattevin, Obadia, et al., 2012). Bacteremia due to invasive procedures in the dental clinic rarely cause I.E., according to the American Heart Association (Baddour et al., 2015), the European Society of Cardiology (Habib et al., 2015), the French Society of Cardiology (Millot et al., 2017) and by others (Wang, Gaca, & Chu, 2018). These authoritative sources instead endorse the perspective that “everyday bacteremia” is a far more important risk factor for contracting I.E. than sporadic invasive procedures in a dental clinic, a hypothesis that is not new (Roberts, 1999). Admittedly, the evidence basis for a relationship between oral health status and bacteremia modulated by the extent of ill health has been ambiguous until recently (Tomás, Diz, Tobías, Scully, & Donos, 2012). However, recent unique data originating from the French national health insurance administrative data comprising 138876 patients with prosthetic heart valves support the explicit statement by the investigators: “ *… most cases of infective endocarditis are due to everyday life bacteraemia*.” (Tubiana et al., 2017).

A senior student relayed recently to undersigned a puzzling exam question in oral surgery. They should provide a rational dental treatment plan for a debilitated elderly male with artificial heart valves and a reduced dentition compromised by multiple sites of apical and marginal periodontitis about to undergo extensive general surgery. The student had learned that the “correct answer” was not only to extract the remaining teeth to reduce the risk of contracting I.E., but also to provide new dental implants. Understandably, the student wished to learn about any evidence to clarify the “correct answer”.

Several thoughts emerged while listening to the student. When a need arises for an acute surgical operation the quickest way to eliminate infection foci associated with a tooth is by first removing the tooth and then the related infected tissue. That the evidence for a rapid and invasive “dental clearance” practice just before a planned cardiovascular surgery is limited and that such practice is not necessarily beneficial for the patient is another matter (Cotti et al., 2017).

On the other hand, the idea of replacing the bad teeth with implants caused me concerns. Both periodontal and peri‐implant infections develop secondary to the formation of pathogenic biofilm, and the pathogenic microorganism do not differ markedly (Lafaurie et al., 2017). If a dentition display accumulated damage caused by insufficient oral hygiene, why can one assume that this situation will improve after providing the patient with dental implants? Moreover, if the inadequate oral hygiene persists, what is the likelihood that a peri‐implant infection will develop eventually? Finally, and most importantly, while teeth with apical or marginal infections can be conserved successfully and with high predictability, unfortunately, the same does not apply to peri‐implant infections.

Hesitant about whether some new evidence had surfaced recently, I embarked on a quest to identify potential scientific literature, albeit with a tiny yield. Although the lack of RCTs was not surprising, it is odd that there is an almost total lack of clinical evidence to support any recommendation. Only one clinical study could be identified describing a small study population (*n* = 13) over 17 years (Findler, Chackartchi, & Regev, 2014). Although the findings were assuring, the study methodology and retrospective design raise a concern of potential reporting bias. Another study with a tentatively promising title: *“Patterns of mortality of patients treated with dental implants.*..” (Jemt, Kowar, Nilsson, & Stenport, 2015) did not mention endocarditis. Hence, the next initiative was to scrutinize the literature for guidelines and expert opinions and perhaps, in the end, rely only on following the reasoning of good old William Osler and Abraham Flexner.

Only the ESC 2015, to a limited extent, and the French 2017 guidelines discuss the use of dental implants. The ESC 2015 guideline contains a comment that the use of dental implants raises concerns due to a foreign material at the interface between the buccal cavity and blood, but state also that there is no current evidence to contraindicate dental implants in all patients at risk for I.E. Nonetheless, a recommendation is that this indication should be discussed on a case‐by‐case basis and that the patient should be informed of the uncertainties and the need for close follow‐up. The French 2017 guideline provides a more thorough critical appraisal of the pros and cons of placing dental implants in patients at risk of I.E. In short, dental implants may be recommended if the estimated risk of future peri‐implant infection is minimal, that the patient demonstrates meticulous oral hygiene, and that the patient complies with an oral health status control twice per year.

In sum, given current evidence, the exam question describes a case scenario where it would appear that placing dental implants would likely have increased the risk of I.E. This appraisal is not a denunciation of the use of dental implants in patients at risk of I.E. A critical aspect is that care providers must not only provide important information to the patient but also ensure that he or she has understood its full impact so future ill health may be avoided. It is quite astonishing to read deep into the article that reports the data from the large French cross‐sectional study (Tubiana et al., 2017) that only 50% of the patients received dental care during a mean follow‐up of 1.7 years after being diagnosed with I.E. despite a recommendation to visit a dentist twice a year. Taking for granted that most I.E. patients desire to remain alive, what is the reason for this inexplicable statistic? Is it perhaps miscommunication, under‐communication, poor access to oral care or poor financial coverage by national and private health insurance? We should always remember that the patient and the provider differ with regard to an interpretation of estimated risk,i.e., the product of the probability of an (adverse) event and the consequences of an (adverse) event. A unique case description pertinent to the question is the patient with a history of multiple I.E. who elects to remove all teeth and forego dental implants in recognition of the potential of harboring bacterial species associated with I.E. in the peri‐implant sulcus formation (Dhima, Salinas, & Koka, 2011). I believe this paper highlights nicely the obligation we have as health professionals to provide honest and unbiased information as a basis for shared decision‐making.

Lastly, be also reminded when we provide any recommendations to our students and patients of the quote by a famous French writer and philosopher, “*Doubt is not a pleasant condition, but certainty is an absurd one*” (Voltaire, 1694–1778).
